# Ketamine independently modulated power and phase-coupling of theta oscillations in *Sp4* hypomorphic mice

**DOI:** 10.1371/journal.pone.0193446

**Published:** 2018-03-07

**Authors:** Xin Wang, António Pinto-Duarte, M. Margarita Behrens, Xianjin Zhou, Terrence J. Sejnowski

**Affiliations:** 1 Howard Hughes Medical Institute, the Salk Institute for Biological Studies, La Jolla, California, United States of America; 2 Department of Psychiatry, University of California at San Diego, La Jolla, California, United States of America; 3 Division of Biology, University of California at San Diego, La Jolla, California, United States of America; University of Oxford, UNITED KINGDOM

## Abstract

Reduced expression of Sp4, the murine homolog of human SP4, a risk gene of multiple psychiatric disorders, led to N-methyl-D-aspartate (NMDA) hypofunction in mice, producing behavioral phenotypes reminiscent of schizophrenia, including hypersensitivity to ketamine. As accumulating evidence on molecular mechanisms and behavioral phenotypes established *Sp4* hypomorphism as a promising animal model, systems-level neural circuit mechanisms of *Sp4* hypomorphism, especially network dynamics underlying cognitive functions, remain poorly understood. We attempted to close this gap in knowledge in the present study by recording multi-channel epidural electroencephalogram (EEG) from awake behaving wildtype and *Sp4* hypomorphic mice. We characterized cortical theta-band power and phase-coupling phenotypes, a known neural circuit substrate underlying cognitive functions, and further studied the effects of a subanesthetic dosage of ketamine on theta abnormalities unique to *Sp4* hypomorphism. *Sp4* hypomorphic mice had markedly elevated theta power localized frontally and parietally, a more pronounced theta phase progression along the neuraxis, and a stronger frontal-parietal theta coupling. Acute subanesthetic ketamine did not affect theta power in wildtype animals but significantly reduced it in *Sp4* hypomorphic mice, nearly completely neutralizing their excessive frontal/parietal theta power. Ketamine did not significantly alter cortical theta phase progression in either wildtype or *Sp4* hypomorphic animals, but significantly strengthened cortical theta phase-coupling in wildtype, but not in *Sp4* hypomorphic animals. Our results suggested that the resting-state phenotypes of cortical theta oscillations unique to *Sp4* hypomorphic mice closely mimicked a schizophrenic endophenotype. Further, ketamine independently modulated *Sp4* hypomorphic anomalies in theta power and phase-coupling, suggesting separate underlying neural circuit mechanisms.

## Introduction

SP4, a transcription factor targeting GC-rich sequences around the promoters of numerous genes [1], was found to be associated with schizophrenia, bipolar disorder and major depression [2–5]. SP4 gene was also reported to be sporadically deleted in schizophrenia patients [6,7] and had reduced expression in postmortem brains of bipolar patients [8].

Sp4, the murine homolog of human SP4, was found to be selectively expressed in neurons [9,10] and it played an important role in the development of hippocampus [11]. *Sp4* hypomorphic mice had reduced NMDAR1 expression throughout the brain [7,12] consistent with postmortem analysis of schizophrenic brains [13–15], and displayed a host of behavioral phenotypes endophenotypic of schizophrenia and other psychiatric disorders [7,10,11,16], including deficit in prepulse inhibition (PPI) [10] and, as we recently reported, hypersensitivity to ketamine [16]. Ketamine, a non-competitive NMDAR antagonist, is known to induce schizophrenia-like behavioral phenotypes in healthy subjects and exacerbate symptoms in patients [17–20], which is in line with the findings that patients with anti-NMDAR encephalitis developed schizophrenia-like cognitive symptoms [21,22]. Consistent with the hypoglutamatergic hypothesis that schizophrenia is associated with impaired NMDA transmission, therefore, the *Sp4* hypomorphic mouse presents a compelling animal model of the disorder in that it uniquely aggregates multiple validities: genetic (a risk gene), neurochemical (NMDAR hypofunction), behavioral (PPI deficits, etc.) and pharmacological (ketamine hypersensitivity).

As knowledge of Sp4-related mechanisms continued to accumulate on these multiple fronts [7,10–12,16,23,24], systems-level characteristics of the *Sp4* hypomorphic mouse, especially neural circuit dynamics associated with cognitive functions, remain poorly understood. Thus, it calls for systems neuroscientific efforts to identify intermediate neurophysiological phenotypes of the model. In the current study, we made such an attempt in order to close this gap in knowledge.

Specifically, we explored *Sp4* hypomorphism-associated phenotypes of cortical theta oscillations and their sensitivity to ketamine. Rhythmic activities at multiple time scales underlie important cognitive functions [25–27], while aberrant neural circuit dynamics and cognitive impairments often coexist in psychiatric disorders [28]. Theta-band oscillations, in particular, are known to coordinate activities amongst different brain regions, which were necessary for memory functions [26,27,29–32]. To date, an increasing number of studies using electrophysiological recording techniques have reported anomalous prefrontal and hippocampal theta-band activities and their synchrony in a number of animal models of schizophrenia [33–37].

*In vivo* electrophysiological investigations using mouse models are often limited by technical difficulties due to a lack of powerful methods. In this study, we took advantage of a novel multi-channel epidural electroencephalographic method of high precision and reliability, which we recently developed [38], and for the first time, applied it to studying circuit dynamic phenotypes of *Sp4* hypomorphic mice.

## Materials and methods

All procedures were conducted in accordance with guidelines of the National Institutes of Health and were approved by the Institutional Animal Care and Use Committee (IACUC) at the Salk Institute.

### Surgery

**Fig 1:**
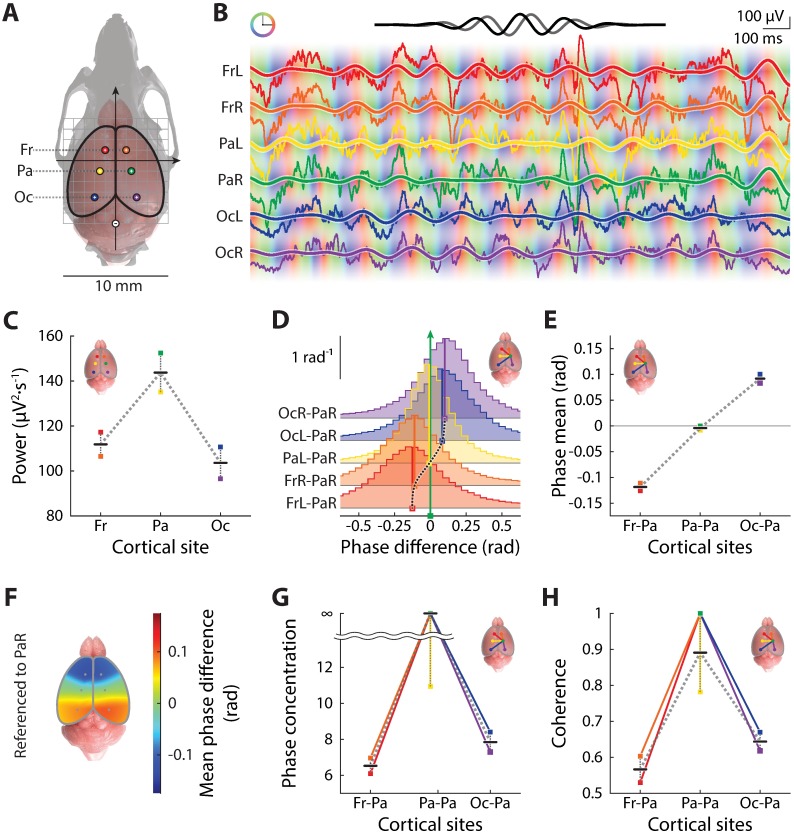
Characteristics of cortical theta power and phase-coupling in an adult mouse. (A) Our multi-channel epidural EEG recording setup illustrated by a top-view of the positioning of electrodes on the surface of the cerebral cortex; for technical details see [38]. Recording electrodes are represented by colored circles (color code consistent in the rest of the Figure) labeled with plus signs and the reference electrode a minus sign, all marked on the surface of the brain (pink) masked by a transparent skull (light gray; adapted from the Henderson 3D surgical atlas of adult mice [56]) drawn to scale. A millimeter grid (gray) is overlaid on top of the brain and solid arrows represent the major anatomical axes, with the origin at the position of bregma. Descriptors of pairs of recording sites along neuraxis, Fr (frontal), Pa (parietal) and Oc (occipital), are labeled on the side. (B) Sample raw EEG recordings (thin traces) from a behaving, adult male C57BL/6J mouse and corresponding theta waves (thick curves) estimated by a complex Morlet transform with a Morlet wavelet centered at 6 Hz (top, real part in black and imaginary in gray). Instantaneous theta phases are color-coded (color ring inset on the top-left) as the background along the traces. (C) Average theta power along the neuraxis on the cortical surface as estimated by the instantaneous amplitudes of the Morlet transform. Colored square symbols represent recording sites, average levels at specific antero-posterior coordinates are marked by horizontal lines, and the dotted line the trend along the neuraxis, inset showing the locations of the recording sites, convention same for the rest of the figure. (D) Distributions of theta phase differences between each recording site and a reference identified as parietal-right (as illustrated by the inset at the top right corner, and labels on the left), estimated as histograms (colored staircase plots). Mean of the distributions are marked by vertical lines with the same color code. A gradient of mean theta phase along the neuraxis is evident as illustrated by the dotted trend line. (E) The gradient of mean theta phase difference along the neuraxis on the cortical surface. (F) A pseudo-color spatial map of interpolated mean theta phase differences over the cerebral cortical surface. (G) Theta phase coupling strength quantified by phase concentration (see Materials and methods). (H) Theta phase coupling strength quantified by spectral coherence (see Materials and methods).

The surgical procedures were exactly the same as previously described [38]. During a 15-minute-long procedure under isoflurane anesthesia, a multi-electrode epidural implant device was introduced and fixed on the skull of an adult mouse, in a stereotaxic apparatus. Six epidural EEG electrodes sampled the surface of the cerebral cortex in a bilaterally symmetric manner, all referenced to an epidural electrode placed in the midline on top of cerebellum (Fig 1A). These three pairs of recording sites long the neuraxis were labeled as frontal (Fr), parietal (Pa) and occipital (Oc). Note that these terms were not used in a strict anatomical sense; this is because, though the Fr and Pa recording sites were on the frontal and parietal crania, respectively, Oc sites were in fact on parietal (rather than occipital) cranium as well. Nonetheless, the Oc sites targeted the visual cortex in mice, which is functionally analogous to the human occipital cortex. For details see previous method paper [38] and S5 Fig.

### Recording

Recording was performed after post-operative recovery (1–2 days). Two types of recording sessions were designed for this study. The first type is a simple 1-hour-long session of recording from freely behaving animals; the resulting data were used to study the characteristic theta oscillation phenotypes. The second type is a 3-hour-long session divided into three 1-hour-long segments, separated by two intraperitoneal (i.p.) injections at the passing of each hour, the first injection being of a saline vehicle and the second of a subanesthetic dosage (50 mg/kg) of ketamine; the resulting data were used to study the effects of ketamine on theta oscillations. The time windows of injections were typically 5–10 minutes in length, thus leaving 50–55 minutes of EEG recording for each segment.

All recording sessions were conducted during the light phase of the circadian cycle. Before each recording session, the animal subject was habituated to a square, transparent acrylic recording chamber 25 cm by 25 cm in size [38]. Throughout an entire recording session, EEG signals were continuously recorded with open filter setting, sampled at 1 kHz and digitized at 16-bit precision. For details see previous method paper [38].

### Data analysis

First, time windows of i.p. injections and extraneous values (defined as absolute value larger than 1 mV) within raw EEG time series were rejected, as preprocessing. Let a preprocessed EEG signal be denoted *x*(*t*) (e.g. Fig 1B, thin traces). Next, complex Morlet transform was applied to EEG signal *x*(*t*) using the following wavelet

$$\psi (t;f)=\pi^{-\frac{1}{4}}f^{\frac{1}{2}}{\left(1+e^{-4\pi^{2}}-2e^{-3\pi^{2}}\right)}^{-\frac{1}{2}}e^{-\frac{1}{2}{f^{2}t}^{2}}(e^{i2\pi ft}-e^{-2\pi^{2}}),$$

where central frequency *f* = 6 Hz, resulting in a complex signal *ξ*(*t*) = [*x* * *ψ*](*t*), representing the theta-band activity (Fig 1B, thick curves, showing real part). See S2 and S3 Figs for full spectra of reported physiological variables.

Instantaneous power was then estimated as

$$p_{x}(t)=\xi (t)\overset{-}{\xi }(t),$$

($\overset{-}{\xi }$ being the complex conjugate of *ξ*) whose time average 〈*p*_*x*_〉 (angular brackets denote time-averaging over the recording period of interest) was used to quantify overall power of theta activities (Fig 1C).

For simultaneously recorded EEG signals *x*(*t*) and *y*(*t*)from two cortical sites, let *ξ*(*t*) = [*x* * *ψ*](*t*) and *η*(*t*) = [*y* * *ψ*](*t*) be the corresponding theta-band complex signals. Then the instantaneous theta phase difference between *x*(*t*) and *y*(*t*) was computed as (Fig 1D)

$$\phi_{x-y}(t)=\text{Im}\;\text{Log}\frac{\xi (t)}{\eta (t)}.$$

In order to characterize theta phase coupling between *x*(*t*) and *y*(*t*), two descriptive statistics of the distribution of *ϕ*_*x*−*y*_, namely the circular mean ${\hat{\theta }}_{x-y}$ (Fig 1E) and circular concentration ${\hat{\kappa }}_{x-y}$ (Fig 1G), were computed. These were defined by

$${\hat{\theta }}_{x-y}=\text{arg}\langle e^{i\phi_{x-y}}\rangle ,$$

$$\frac{I_{1}({\hat{\kappa }}_{x-y})}{I_{0}({\hat{\kappa }}_{x-y})}=\langle {\left|e^{i\phi_{x-y}}\right|}^{2}\rangle ,$$

where *I*_*n*_(·) is the modified Bessel function of the *n*-th order. Analogous to mean and variance in non-circular cases, the circular mean represented average theta phase lag between *x*(*t*)and *y*(*t*), whereas the circular concentration described how strongly *x*(*t*) and *y*(*t*) were phase-locked (or synchronized) at theta rhythm. Thus, we used ${\hat{\theta }}_{x-y}$ and ${\hat{\kappa }}_{x-y}$ as metrics to quantitatively describe theta phase-coupling across cortical recording sites.

Finally, we also quantified a commonly used metric for phase-coupling strength, i.e. spectral coherence (Fig 1H), which is related to power and circular statistics of phase differences defined above. The theta coherence between *x*(*t*) and *y*(*t*) was estimated as

$$c_{x-y}=\frac{{\left|\langle \xi \overset{-}{\eta }\rangle \right|}^{2}}{\langle \xi \overset{-}{\xi }\rangle \langle \eta \overset{-}{\eta }\rangle }.$$

For all data in this study, theta phase concentration ${\hat{\kappa }}_{x-y}$ and theta coherence *c*_*x*−*y*_ were highly correlated (e.g. Fig 1G and 1H), and thus either of these was used as a proxy of the strength of theta phase-coupling.

For all statistical tests of physiological variables between groups of interest in this paper, we reported p-values of non-parametric Mann-Whitney-Wilcoxon tests (though, due to general Gaussianity of most data in this study, Student’s t-tests yielded qualitatively the same results for all hypothesis tests reported here). We used p < 0.05 as a criterion for statistical significance.

Data analyses were performed using MATLAB (Mathworks, Natick MA) and a circular statistics toolbox [39].

## Results

We recorded from 19 mice, including 9 wildtype and 9 *Sp4* hypomorphic mice (both were the F1 generation of 129S and Black Swiss genetic backgrounds) at 5–6 months of age, and additionally one 3-month-old C57BL/6J mouse for a preliminary study. In all experiments the animals were freely exploring without explicit tasks (for a control for behavioral states see S1 Fig and S1 Table). Using a novel multi-channel epidural EEG technique [38], here we, for the first time, quantified cortical theta power and phase-coupling phenotypes associated with *Sp4* hypomorphism in a systematic manner.

### Characteristics of cortical theta power and phase-coupling in mice

Taking advantage of the new technique, we established a number of key phenotypic characteristics of cortical theta power and phase-coupling in adult mice. Here we use recording data from an adult male C57BL/6J mouse to illustrate the typical murine phenotype of cortical theta oscillations (Fig 1). Theta-band activities were extracted by means of a complex Morlet transform with a Morlet function at 6 Hz and of central frequency 1 (Fig 1B, see Materials and methods).

Theta power had a characteristic distribution over cortex: it was highest at parietal recording sites, and tapered down going either anteriorly or posteriorly (Fig 1C), a trend consistent with these cortical recording sites’ anatomical proximity to the hippocampal formation.

Theta phase also had a systematic distribution over the cortical surface (Fig 1D–1F). When referenced to a parietal channel (PaR), relative theta phases of frontal channels had negative means and those of occipital channels had positive means (Fig 1D and 1E); i.e. frontal theta rhythms on average lagged behind the parietal, which in turn, lagged behind the occipital. Such a phase gradient suggested a pronounced posterior-to-anterior theta phase progression along the neuraxis (Fig 1F).

Besides the means of the distributions of theta phase differences, the widths of these distributions (Fig 1D) were another meaningful statistic that measured how strongly theta rhythms were phase-locked, or synchronized. Thus, we quantified strength of phase coupling by phase concentration (Fig 1G, see Materials and methods), as well as conventional spectral coherence (Fig 1H, see Materials and methods).

Notably, both the power and phase-coupling of theta oscillations demonstrated bilateral symmetry in mice. Hence, in the subsequent analyses, we pooled data from both sides whenever possible.

### Theta power phenotypes characteristic of *Sp4* hypomorphism

**Fig 2:**
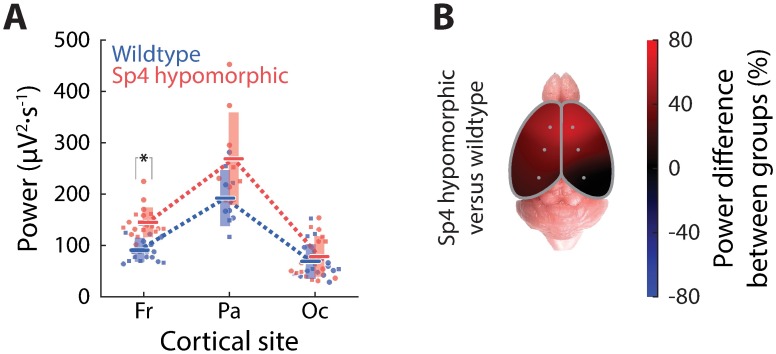
Theta power phenotypes characteristic of *Sp4* hypomorphism. This figure contains data from 9 wildtype and 9 *Sp4* hypomorphic mice; recording data from both sides were pooled whenever possible, thanks to bilateral symmetry; same convention followed throughout the paper. (A) Average theta power along the neuraxis plotted for wildtype (blue) and for *Sp4* hypomorphic (red) animals, similar to Fig 1C. Single recording site from an animal is represented by a symbol (circles for female and squares for male, though no gender difference was found in this study) forming a bee-swarm chart; group averages are marked by horizontal lines and one standard deviation error by light-colored vertical bars; dotted lines show trends along the neuraxis. Asterisks indicate statistically significant differences (see Materials and methods for technical details and see Results for p-values). Conventions remain the same throughout the paper. (B) A pseudo-color spatial map over the cerebral cortical surface showing the percentage differences of theta power in *Sp4* hypomorphic as compared to wildtype animals.

First, we asked whether *Sp4* hypomorphism led to altered cortical theta power. To address this question, we quantified theta power recorded from various cortical sites in wildtype versus *Sp4* hypomorphic animals (Fig 2A). We noted a considerable dependence of measured theta activities on mouse strains (see S4 Fig) and thusly used wildtype control groups of the same genetic background.

While both wildtype and *Sp4* hypomorphic animals displayed the typical cortical distribution of theta power (cf. Fig 1C), *Sp4* hypomorphics had higher theta power than wildtype animals in general, and this difference in theta power was dependent on cortical loci. Specifically, theta power, in *Sp4* hypomorphic mice as compared to wildtype (Fig 2A), was significantly higher at frontal sites (p = 7.57×10^−6^), marginally higher at parietal sites (p = 0.0503), and statistically identical at occipital sites (p = 0.716). On average, *Sp4* hypomorphism led to about 40% higher theta power and this increase of theta activity was specifically localized frontally and parietally (Fig 2B).

### Theta phase-coupling phenotypes characteristic of *Sp4* hypomorphism

Next, we questioned whether *Sp4* hypomorphism also led to characteristic phenotypes in cortical theta phase-coupling.

**Fig 3:**
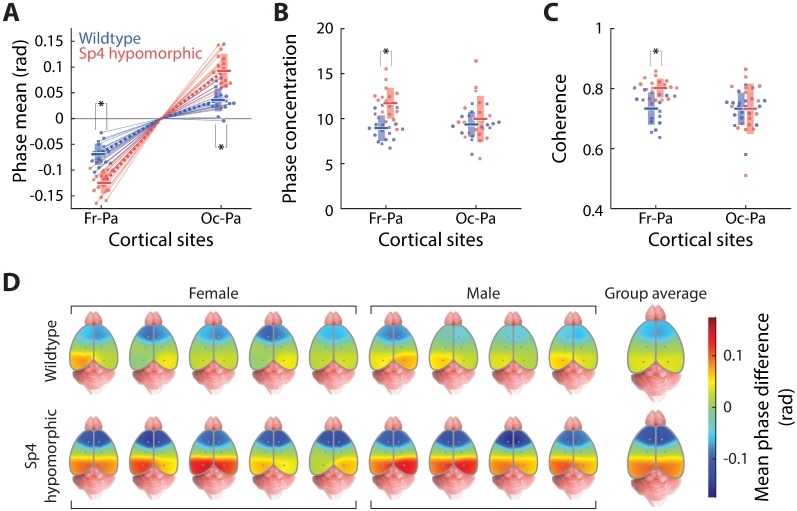
Theta phase-coupling phenotypes characteristic of *Sp4* hypomorphism. This Fig contains data from 9 wildtype and 9 *Sp4* hypomorphic mice. (A) The gradient of mean theta phase difference along the neuraxis on the cortical surface, similar as in Fig 1E, convention same as previous. (B) Theta phase concentrations of frontal-parietal and occipital-parietal pairs. (C) Theta spectral coherences of frontal-parietal and occipital-parietal pairs. (D) Pseudo-color spatial maps of mean theta phase differences over the cerebral cortical surface (similar as Fig 1F) for each animal, wildtype animals organized in the top row and *Sp4* hypomorphic the bottom row. Population averages of the two genotype groups are shown on the right.

As suggested by quantifications of the mean theta phase difference between recording sites (Fig 3A, cf. Fig 1E), *Sp4* hypomorphic mice had significantly higher (p = 6.26×10^−7^ for frontal-parietal phase differences and p = 2.09×10^−5^ for occipital-parietal phase differences) cortical theta phase gradients along the neuraxis than wildtype animals (Fig 3A and 3D), suggesting a roughly two-fold slower theta phase progression along the neuraxis associated with *Sp4* hypomorphism (Fig 3D). It should be noted that *Sp4* hypomorphic mice did not have distinctive gross anatomical features as compared to wildtype animals, and we did not find significant differences in brain size for these animals; thus, the distinct theta phase gradient observed here was not simply due to aberrant brain size or shape, but a true physiological phenomenon, though micro-structural changes not observable at the macroscopic anatomical level cannot be ruled out.

Furthermore, a significantly stronger theta phase-coupling was observed between frontal and parietal loci (p = 4.79×10^−5^ for theta phase concentration and p = 4.09×10^−5^ for theta spectral coherence), whereas phase synchrony between occipital and parietal was statistically identical (p = 0.669 for theta phase concentration and p = 0.962 for theta spectral coherence) between wildtype and *Sp4* hypomorphic animals (Fig 3B and 3C).

### Effects of acute, subanesthetic ketamine on theta power phenotypes

**Fig 4:**
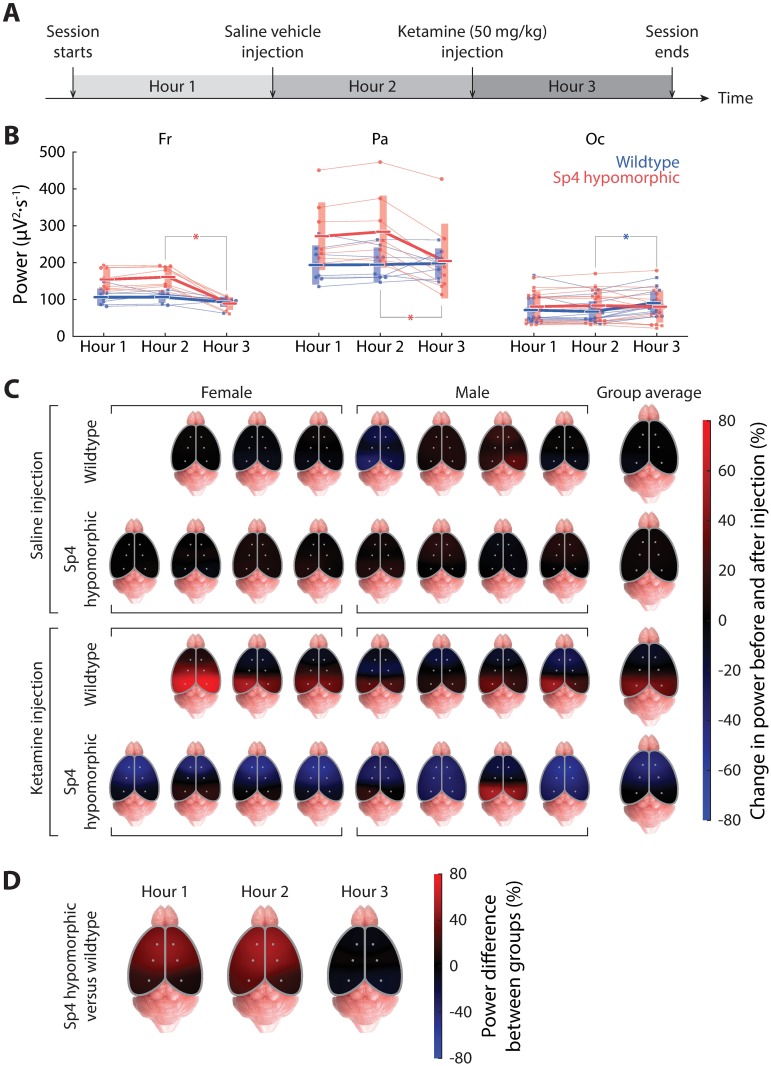
Effects of acute, subanesthetic ketamine on theta power phenotypes in wildtype and *Sp4* hypomorphic mice. This figure contains data from 7 wildtype and 8 *Sp4* hypomorphic mice. (A) A timeline showing the experimental design. Each recording session was 3-hour-long with intraperitoneal injections of a saline vehicle and of a subanesthetic dosage (50 mg/kg) of ketamine administered at the passing of hours. Each hour of the recording session was analyzed separately for baseline, and for effects of the saline and ketamine injections. (B) Temporal evolution of average theta power during the recording session for frontal (left), parietal (middle) and occipital (right) recording sites. Color code and graphic symbols follow same conventions as previous, except that the solid lines (thin lines for individuals and thick ones for group averages) represent changes over time. (C) Pseudo-color spatial maps of percentage changes in theta power after versus before an injection (top two rows: saline; bottom two rows: ketamine). Each animal is shown on the left with genotype groups organized into rows; group averages are plotted on the right. (D) Pseudo-color cortical map showing the percentage differences of theta power in *Sp4* hypomorphic as compared to wildtype animals (similar as Fig 2B) for the three segments of the recording session.

In order to study how acute, subanesthetic ketamine modified theta power and phase-coupling phenotypes in wildtype and *Sp4* hypomorphic animals, we designed a recording session consisting of three 1-hour-long segments separated by i.p. injections of a saline vehicle and a subanesthetic dosage (50 mg/kg) of ketamine (Fig 4A). The three segments were analyzed separately for conditions of baseline, effects of saline and effects of ketamine. Changes in specific physiological variables from Hour 1 to Hour 2 were attributed to effects of saline injection, and changes from Hour 2 to Hour 3 to those of ketamine.

We did not find injections of saline vehicles induced any statistically significant changes in theta power from all cortical recording sites in both genotype groups (Fig 4B and 4C top). Ketamine injections, in contrast, induced significant changes in theta power dependent on the cortical recording sites. These effects on theta power included: a highly significant reduction at frontal sites (p = 1.55×10^−4^) and a marginally significant reduction at parietal sites (p = 0.0499) in *Sp4* hypomorphic animals, as well as a slight but significant increase at occipital sites (p = 0.0203) in wildtype animals (Fig 4B). The locus-specificity of ketamine-induced changes in cortical theta power can be better appreciated by the spatial maps of changes (Fig 4B bottom).

In sum, the net effects of ketamine were most prominently a frontally localized reduction of theta power in *Sp4* hypomorphic, but not in wildtype, animals. Since *Sp4* hypomorphics, to begin with, had a roughly 40% higher theta power profile, also frontally/parietally concentrated (Fig 2B), acute ketamine effectually neutralized this excessive frontal/parietal theta power, and brought the overall cortical profile of theta power down to one that was practically indistinguishable from that of wildtype animals (Fig 4B and 4D).

### Effects of acute, subanesthetic ketamine on theta phase-coupling phenotypes

Unlike its effects on theta power, ketamine modulated cortical theta phase-coupling in a remarkably distinct manner.

**Fig 5:**
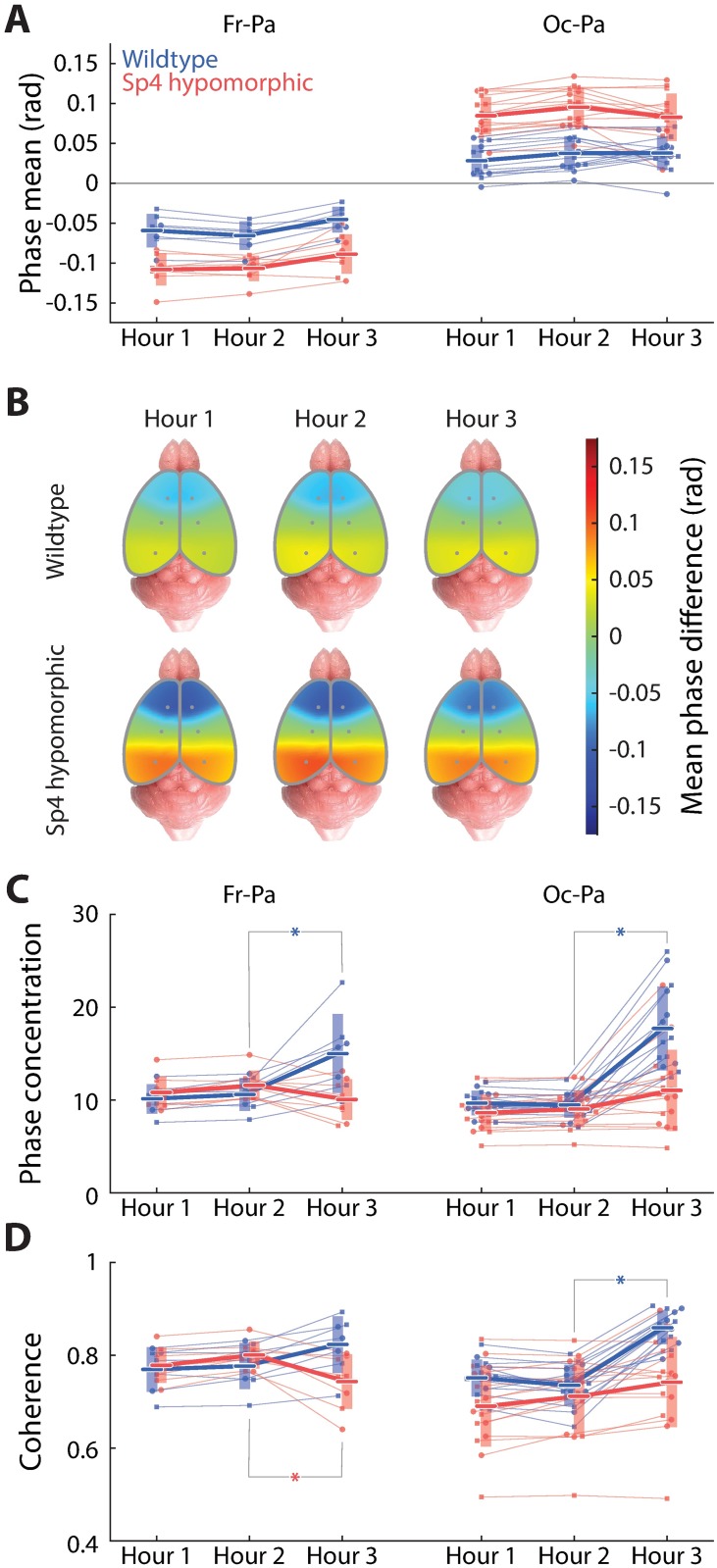
Effects of acute, subanesthetic ketamine on theta phase-coupling phenotypes in wildtype and *Sp4* hypomorphic mice. This figure contains data from 7 wildtype and 8 *Sp4* hypomorphic mice. (A) Temporal evolution of mean theta phase differences during the recording session for frontal-parietal (left) and occipital-parietal (right) pairs of cortical sites. (B) Pseudo-color cortical maps of mean theta phase differences (similar as Fig 1F) for wildtype (top row) and *Sp4* hypomorphic (bottom row) animals, and for each of the three segments of the recording session (columns). (C) Temporal evolution of theta phase concentrations during the recording session. (D) Temporal evolution of theta spectral coherences during the recording session.

First, ketamine did not significantly affect the magnitudes of the theta phase gradients along the neuraxis in either wildtype or *Sp4* hypomorphic mice (Fig 5A and 5B). Only a slight, statistically insignificant reduction of theta phase gradient was resulted from ketamine injections (Fig 5A); nevertheless, the roughly two-fold slower theta phase progression along the neuraxis observed in *Sp4* hypomorphic animals (Fig 3D) was unchanged after ketamine injection (Fig 5B). Thus, the cortical theta phase progression phenotypes in either wildtype or *Sp4* hypomorphic animals was completely insensitive to subanesthetic ketamine.

In contrast, however, ketamine had its unique effects on the strengths of cortical theta phase-coupling in these animals (Fig 5C and 5D). Specifically, in wildtype animals, ketamine marginally strengthened frontal-parietal theta phase-coupling (p = 0.0262 for phase concentration and p = 0.126 for spectral coherence, respectively; Fig 5C and 5D) and significantly strengthened occipital-parietal theta phase-coupling (p = 7.47×10^−7^ for phase concentration and p = 9.25×10^−7^ for spectral coherence, respectively; Fig 5C and 5D). In *Sp4* hypomorphic animals, on the contrary, ketamine marginally weakened frontal-parietal theta phase-coupling (p = 0.235 for phase concentration and p = 0.0206 for spectral coherence, respectively; Fig 5C and 5D) but slightly strengthened occipital-parietal theta phase-coupling (statistically insignificant; Fig 5C and 5D). Note that the significantly stronger frontal-parietal coupling in *Sp4* hypomorphic animals described earlier (Fig 3B and 3C) did not turn out to reach significance level in these recordings, probably due to a lack of power as the sample size was smaller than half of the previous case (data from only one side in injection studies versus both sides in preliminary); nevertheless, this sample was large enough to identify the abovementioned ketamine-induced effects on theta phase-coupling strengths, thanks to their extraordinarily pronounced magnitudes.

In sum, while ketamine had little effects on the distinct magnitudes of cortical theta phase progression in either wildtype and *Sp4* hypomorphic mice, ketamine induced drastically different reactions in terms of theta phase-coupling strength in wildtype versus *Sp4* hypomorphic animals: in particular, ketamine substantially increased cortical theta coupling strength in wildtype animals, whereas *Sp4* hypomorphics were largely insensitive to ketamine-induced change in theta coupling, if not subject to a marginal change in the opposite direction. Consequently, after ketamine injection, wildtype mice displayed much stronger cortical theta phase-coupling than *Sp4* hypomorphics, reversing the trend observed before ketamine injection.

## Discussion

Cortical rhythms in the theta band play an important role in cognitive functions; in particular, theta oscillations are well-established neural substrates underlying working memory [26,27,29–31]. Anomalies involving theta rhythms have been reported in schizophrenic patients [40–54], as well as in a number of animal models of the disorder [33–37]. Recent work also suggested cortical theta oscillations as a potential therapeutic target [55].

In this study, we used a novel multi-channel epidural EEG recording technique [38] to characterize unique cortical theta oscillation phenotypes associated with the *Sp4* hypomorphic mouse model, and examined how they were affected by acute subanesthetic ketamine. Because different mouse strains have different sensitivity to ketamine, we determined the dosage of 50 mg/kg based on dose-response curve mapped by previous work on *Sp4* hypomorphic mice [16,23]. Consistent with these earlier reports, for all animals used in this study, i.p. injection of 50 mg/kg ketamine induced immediate increase in locomotion without any effects of sedation [16], suggesting the dosage used was subanesthetic.

This is a first report on *Sp4* hypomorphic mice’s intermediate neurophysiological phenotypes known to underlie cognitive functions, which, in our opinion, provided a timely complement to the recent advances in the understanding of molecular and behavioral mechanisms associated with hypoglutamatergia in the *Sp4* hypomorphic model [7,10,11,16,23]. Moreover, the high spatial precision of our measurement of cortical theta waves [38] yielded important localization information often hard to obtain in studies using behaving mice.

Our results revealed three distinct aspects of cortical theta phenotype associated with *Sp4* hypomorphism. First, we found that *Sp4* hypomorphic mice had remarkably higher cortical theta power, an about 40% increase from the wildtype level, and such an increase was specifically localized frontally and parietally. Second, we observed a significantly larger cortical theta phase gradient in *Sp4* hypomorphic animals, suggesting a roughly two-fold slower cortical theta phase progression than that in wildtype animals (note that the spatial gradients of theta phase over the cortical surface were significantly lower than recorded from the hippocampus by depth electrodes). Third, we also observed a significantly stronger frontal-parietal theta phase-coupling in *Sp4* hypomorphics.

Multiple studies consistently have reported higher levels of resting theta power in schizophrenic patients than in normal subjects, and in cognitively dysfunctional than functional patients [40,42–44,49–52]. Higher resting theta power was also reported in two rat models of schizophrenia [35,37]. Our data conclusively demonstrated that the *Sp4* hypomorphic mouse model accurately mimics this phenotype. In contrast to the abundance of evidence on schizophrenia-related increased resting (i.e. in a task-free context) theta power reported in the human literature, whether the specific phenotypes of resting-state cortical phase-coupling of theta waves we observed in *Sp4* hypomorphic mice actually corresponded to disease-specific signatures (especially the slower theta phase progression along the neuraxis) lacked previous matching studies in humans, and thusly remains an open question for future studies to explore.

The most striking discovery of the present study was that subanesthetic ketamine affected the three aspects of *Sp4* hypomorphism-associated cortical theta phenotype in a completely independent manner. First, ketamine significantly lowered frontal theta power in *Sp4* hypomorphic, but had no effect on wildtype, animals. Second, ketamine had no effect on cortical theta phase progression in either wildtype or *Sp4* hypomorphic mice. Finally, ketamine significantly strengthened cortical theta phase-coupling in wildtype, but not in *Sp4* hypomorphic, animals.

These results suggest that several entirely different NMDAR-mediated mechanisms might underlie theta power and phase-coupling. Thus, our findings revealed a high level of diversity in neural circuit underpinnings of cortical theta oscillations. These factors likely included complex developmental and physiological processes that led to both structural (e.g. network connectivity) and physiological (e.g. neuronal excitability) alterations in the animal model, thereby displaying different, or sometimes even opposite, sensitivity to ketamine dependent on certain aspects of theta waves. Whether any of these neural circuit traits sensitive or insensitive to ketamine actually corresponded to hypoglutamatergic states of schizophrenia, and how they relate to cognitive function and behavior are important directions of future investigations, for which the current study provides a solid preclinical basis.

A caveat using mouse models is that the animal subjects might experience a diverse range of behavioral states in typical experimental settings, making the conditions considerably different from human experiments in which the subjects are consistently awake and cognitively engaged. To address this problem, we performed all our analyses on a subset of data identified by active time periods in simultaneously recorded electromyograms (EMG), and showed that the observed effects were not confounded by sleep states of the animals (see S1 Text).

As we have shown in previous work, *Sp4* hypomorphic animals were hypersensitive to ketamine in terms of hyperlocomotion and induced gamma activities [16], and recent work using conditional rescue to restore Sp4 expression in specific types of cortical neurons has just started to tease apart specific neural circuit components responsible for the *Sp4* hypomorphic behavioral phenotypes [23]. We anticipate future investigations combining these approaches with the current study would shed light on the questions raised by the present findings.

Finally, the current study used an EEG technique for the mouse with unprecedented convenience, reliability, spatial precision and resolving power [38], and thereby made the *Sp4* hypomorphic mice a compelling case for the benefits of novel electrophysiological methodologies in preclinical psychiatric research using rodent models. As mouse models of psychiatric disorders proliferate, powerful, scalable and standardized mouse EEG techniques become more and more in need to reliably compare and contrast dynamic phenotypes, so as to paint a coherent big picture of various mouse models of a same psychiatric disorder. Our current study provided an initial step toward such a goal.

## Supporting information

S1 Text Supplementary text. (PDF)

S1 Table Results of analyses of whole versus the subset of data of certain wakefulness. (PDF)

S1 Fig Identification of active awake states of high certainty by analyzing simultaneous recorded electromyogram (EMG). (PDF)

S2 Fig Theta-band Morlet and the spatial distribution of theta phases as a function of frequency. (PDF)

S3 Fig Power spectra of frontal, parietal and occipital recording sites. (PDF)

S4 Fig Magnitude of cortical theta phase progression is dependent on genetic background. (PDF)

S5 Fig Functional cortical regions corresponding to the epidural recording sites. (DOCX)
