# Supplementary material for: Ketamine independently modulated power and phase-coupling of theta oscillations in Sp4 hypomorphic mice
Source: PLoS One. 2018 Mar 7;13(3):e0193446. doi: 10.1371/journal.pone.0193446 (PMC5841791; doi:10.1371/journal.pone.0193446)
Supplement: S1 Text — (PDF) [file pone.0193446.s001.pdf]

A. Ruling out the confounding effect of different behavioral states

In order to characterize phenotypes of cortical theta waves associated with *Sp4* hypomorphism (and to explore the effects of subanesthetic ketamine on these phenotypes), we analyzed epidural EEG data from behaving mice during recording sessions that typically lasted between one to three hours. During the entire experiment, the animal subject might have experienced bouts of sleep in addition to awake, behaving states. Thus, different behavioral states introduced a confounding factor to the findings of this study. Specifically, it is under question whether the observed cortical theta phenotypes in *Sp4* hypomorphic mice were a valid model of the similar endophenotypes reported in awake human patients, or a phenomenon associated with sleep states that had nothing to do with awake behaving states of the animal.

To rule out the confounding effect of different behavioral states, we performed further analyses so as to distinguish between sleep and awake states of the animal. Usually, sleep states are reliably characterized by EEG signals. However, identification of sleep bouts using EEG signals is not reasonable in this study because it falls in the trap of circular logic—if the analytical results from the data were used to select the data in the first place, the results might simply be nothing more than a self-fulfilling prophecy. This is especially problematic in this case because here we reported changes in power of relatively slow waves (in the theta range). Fortunately, we recorded simultaneous electromyograms (EMG) in all of our experiments presented in this paper (see [1] for methodological details, Figure S1A). Unlike EEG, EMG could not be used to positively identify sleep bouts, because a lack of voluntary movement does not necessarily mean sleep; however, on the other hand, presence of voluntary muscular activity identified by positive EMG unequivocally suggests wakefulness. Thus, if one singles out a subset of data identified by positive EMG signals, it is then certain that the animal subject was awake during those periods, though they might be only a small fraction of all awake periods. Now, one could reason that, if our findings still held true for this subset data of awake state, we would then effectively rule out the confounding effects of sleep.

To identify positive EMG signals, we used an algorithm introduced by Xu and Adler [2]. We adopted the exact same settings presented in their paper, while EMG baseline was identified by a

whole second of minimal power. In the spirit of the foregoing reasoning, we did not optimize the algorithm to maximize hits, but rather controlled false-alarm rate below p = 0.05, in order to establish a conservative detection mechanism, guaranteeing awake states detected at high confidence level without concerning with potential misses. Figure S1B shows 60 seconds of an example EMG signal and the output of the detection algorithm; the animal was most likely awake during this minute but the algorithm purposefully picked out only those periods of active EMG with high certainty.

Next, we performed all the analyses, exactly as described in the main text, on this identified subset of data of certain wakefulness. We list in Table S1 the p-values of all statistical tests (see main text for technical details) reported in this paper, with results from the entire dataset compared side-by-side with those from the subset of certain wakefulness identified using EMG.

As Table S1 shows, except for minor quantitative differences[*] due to reduced data size, all of the results reported in this paper held true for the subset of data for which the animals were for certain awake. Therefore, we ruled out the possible confounding effect of sleep state.

[*] The most prominent discrepancy is the test for difference in theta phase concentration between the two genotype groups (Table S1, 7th row). This parallels the similar effect seen in ketamine injection data, where a much smaller dataset was analyzed (see main text). Because the identification of awake state with high certainty using EMG typically reduced the data size by several folds, the drop in significance levels of measured effects was most likely due to reduced statistical power, as discussed on the main text. However, all of the key results reported here remained highly significant even with the grossly down-sized dataset of certain wakefulness. Minor, trend-level effects, especially those correspondent to phase-coupling strength (Table S1, last 8 rows) also suffered from a small data size (n = 8).

B. Frequency bands, theta-band Morlet and power/spatial phase as a function of frequency

In the present work, we studied theta-band cortical dynamics, i.e. power and phase, associated with the genetics of *Sp4* hypomorphism and the pharmacology of ketamine. As the theta band spans a non-singular range, typically 4-8 Hz, and phase with regard to a non-singular frequency band is ill-defined, we used a complex Morlet transform. This comes with the choice of a parameter, namely the central frequency, that dictates the bandwidth of the Morlet wavelet. We made all central frequencies 1, and centered the theta-band Morlet at 6 Hz, in the middle of the 4-8 Hz range. The power spectrum of the theta Morlet is plotted in Figure S2A.

Though the observations of theta-band activities in this study are self-contained, it is natural to question whether similar results hold in other frequency bands. This requires a systematic study of spatial structures of power and phase across all frequencies, which is out of the scope of the present work. However, we have already published a systematic study of power as a function of frequency in our previous paper [3], and here, in this supplementary section, we present frequency-dependent spatial phase distributions (Figures S2B, C) based on limited data, i.e. from the same example animal used in the preliminary study shown in Figure 1 of the main text. We also provide power spectra of different recording sites plotted for all individual animals (Figure S3A) as well as their population statistics (Figure S3B). It is clear that the most prominent frequency band of power, spatial phase differences and strengths of coupling is theta, as evident in the peaks of the power spectra (Figure S3), and of the circular statistics as functions of frequency (Figures S2B, C).

C. Magnitude of cortical theta phase progression is dependent on genetic background

It should be noted that the preliminary study presented in Figure 1 of the main text is on a C57BL/6J mouse, which is genetically distinct from the wildtype and *Sp4* hypomorphic animals used in the rest of the study. These are the F1 generation of 129S and Black Swiss genetic backgrounds. Whether the quantified phenotypes (spatial distribution of theta phases in particular) of the C57BL/6J mouse can be considered representative of the wildtype animals, depends on whether these phenotypes under question are the same across these two different strains of mice.

To address this question, we did a systematic quantification of theta phase distributions across three groups: C57BL/6J, wildtype and *Sp4* hypomorphic animals (Figure S4). Our result shows that, despite the same qualitative trend of phase statistics, they are substantially different between C57BL/6J and wildtype mice used in this study, suggesting a strong quantitative dependence of theta phase phenotypes on genetic background. This means that, when studying effects of other factors, such as pharmacological manipulations or developmental stages, having controls of the same genetic background is indispensable in studies of the same kind.

D. Corresponding cortical regions of recording sites

In order to find out the functional cortical regions in which the epidural recording sites were located in this study, we reconstructed a cortical map based on the Paxinos-Franklin mouse brain atlas [4], and overlaid it with the stereotaxically precise recording sites in registration (Figure S5). The occipital recording sites (Oc) were located in the primary visual cortices, the parietal sites (Pa) in the primary somatosensory cortices and the frontal sites (Fr) were on the borders of the primary and secondary motor cortices.

References

1. Wang X, Pinto-Duarte A, Margarita Behrens M, Zhou X, Sejnowski TJ. Characterization of spatio-temporal epidural event-related potentials for mouse models of psychiatric disorders. Sci Rep. Nature Publishing Group; 2015;5: 14964. doi:10.1038/srep14964
2. Xu LXL, Adler A. An improved method for muscle activation detection during gait. Can Conf Electr Comput Eng 2004 (IEEE Cat No04CH37513). 2004;1: 357–360. doi:10.1109/CCECE.2004.1345029
3. Ji B, Wang X, Pinto-Duarte A, Kim M, Caldwell S, Young JW, et al. Prolonged Ketamine Effects in Sp4 Hypomorphic Mice: Mimicking Phenotypes of Schizophrenia. PLoS One. 2013;8. doi:10.1371/journal.pone.0066327
4. Paxinos G, Franklin KBJ. The mouse brain in stereotaxic coordinates. Elsevier Academic Press; 2004.
