# Supplementary material for: Ketamine independently modulated power and phase-coupling of theta oscillations in Sp4 hypomorphic mice
Source: PLoS One. 2018 Mar 7;13(3):e0193446. doi: 10.1371/journal.pone.0193446 (PMC5841791; doi:10.1371/journal.pone.0193446)
Supplement: S1 Table — (PDF) [file pone.0193446.s002.pdf]

Table S1. Results of analyses of whole versus the subset of data of certain wakefulness.

Table cells containing significant values (i.e. $p < 0.05$) are highlighted.

Statistical test		Entire dataset	Subset of wakefulness
Theta power between wildtypes and *Sp4* hypomorphics	Fr	$p = 7.57\times10^{-6}$	$p = 1.06\times10^{-3}$
	Pa	p = 0.0503	p = 0.222
	Oc	p = 0.716	p = 0.987
Theta phase difference between wildtypes and *Sp4* hypomorphics	Fr-Pa	$p = 6.26\times10^{-7}$	$p = 5.49\times10^{-5}$
	Oc-Pa	$p = 2.09\times10^{-5}$	$p = 1.56\times10^{-4}$
Theta phase concentration between wildtypes and *Sp4* hypomorphics	Fr-Pa	$p = 4.79\times10^{-5}$	p = 0.0905
	Oc-Pa	p = 0.669	p = 0.0738
Theta spectral coherence between wildtypes and *Sp4* hypomorphics	Fr-Pa	$p = 1.77\times10^{-4}$	p = 0.0200
	Oc-Pa	p = 0.962	p = 0.304
Ketamine-induced change of theta power in wildtypes	Fr	p = 0.128	p = 0.128
	Pa	p = 1.00	p = 1.00
	Oc	p = 0.0203	p = 0.0326
Ketamine-induced change of theta power in *Sp4* hypomorphics	Fr	$p = 1.55\times10^{-4}$	p = 0.0104
	Pa	p = 0.0499	p = 0.105
	Oc	p = 0.665	p = 0.585
Ketamine-induced changes of theta phase difference in both genotype groups	Fr-Pa<br>Oc-Pa	p > 0.05 for all	p > 0.05 for all
Ketamine-induced change of theta phase concentration in wildtypes	Fr-Pa	p = 0.0262	p = 0.0262
	Oc-Pa	$p = 7.47\times10^{-7}$	$p = 7.47\times10^{-7}$
Ketamine-induced change of theta spectral coherence in wildtypes	Fr-Pa	p = 0.128	p = 0.259
	Oc-Pa	$p = 9.25\times10^{-7}$	$p = 2.62\times10^{-5}$
Ketamine-induced change of theta phase concentration in *Sp4* hypomorphics	Fr-Pa	p = 0.0207	p = 0.721
	Oc-Pa	p = 0.137	p = 0.0438
Ketamine-induced change of theta spectral coherence in *Sp4* hypomorphics	Fr-Pa	p = 0.0207	p = 0.721
	Oc-Pa	p = 0.235	p = 0.137
