# Supplementary material for: Ketamine independently modulated power and phase-coupling of theta oscillations in Sp4 hypomorphic mice
Source: PLoS One. 2018 Mar 7;13(3):e0193446. doi: 10.1371/journal.pone.0193446 (PMC5841791; doi:10.1371/journal.pone.0193446)
Supplement: S1 Fig — (PDF) [file pone.0193446.s003.pdf]

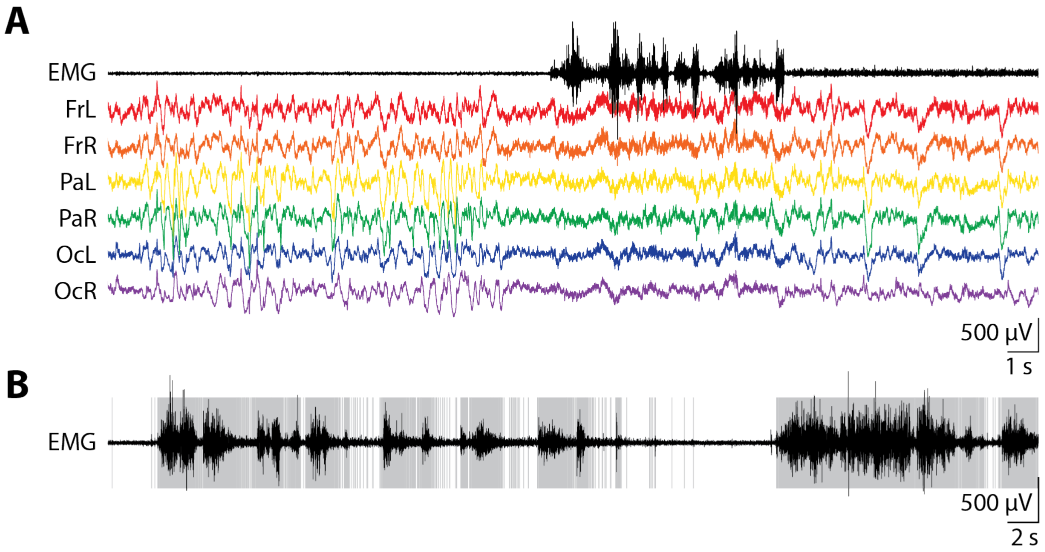

Figure S1. **Identification of active awake states of high certainty by analyzing simultaneous recorded electromyogram (EMG).** (A) An adapted and modified version of Figure 2A from [1], showing simultaneously recorded EMG with EEG. A transition from sleep to wakefulness is present in the middle of this clip. (B) Trace of the EMG signal in a 60-second time window recorded during experiment. The detected periods of active EMG as output of the Xu-Adler algorithm [2] are illustrated by gray shades. All data shown in this figure were obtained from the same animal as in Figure 1 of the main text.
