# Supplementary material for: Ketamine independently modulated power and phase-coupling of theta oscillations in Sp4 hypomorphic mice
Source: PLoS One. 2018 Mar 7;13(3):e0193446. doi: 10.1371/journal.pone.0193446 (PMC5841791; doi:10.1371/journal.pone.0193446)
Supplement: S2 Fig — (PDF) [file pone.0193446.s004.pdf]

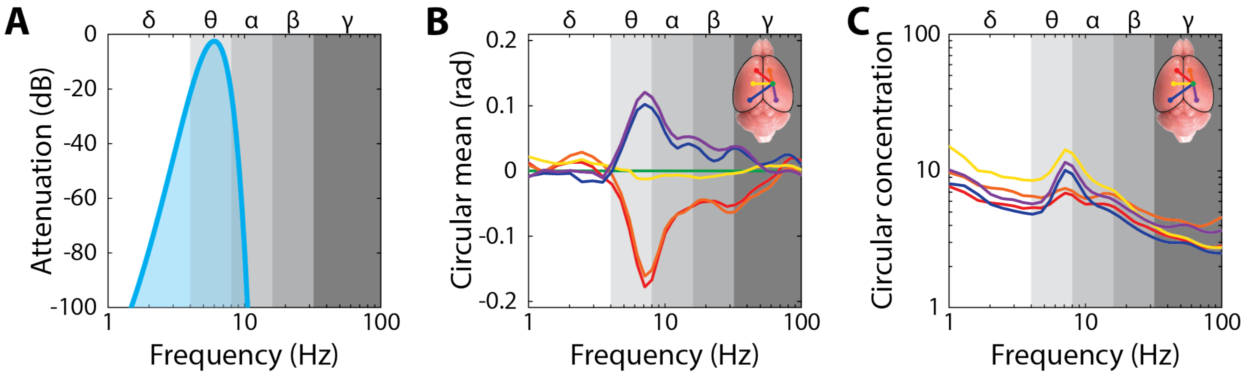

Figure S2. **Theta-band Morlet and the spatial distribution of theta phases as a function of frequency.** (A) Power spectrum (cyan) of the Morlet wavelet used in the present study (centered at 6 Hz). Frequency bands are marked by different shades of gray and specified by Greek letters on the top. (B, C) First- and second-order descriptive statistics (i.e. circular mean and concentration) of spatial phase differences as functions of frequency (estimated from complex Morlet transform, see Materials and Methods). All phase differences are referenced to PaR (see insets), consistent with the main text. Data are from the same animal as in Figure 1 of the main text.
