# Supplementary material for: Ketamine independently modulated power and phase-coupling of theta oscillations in Sp4 hypomorphic mice
Source: PLoS One. 2018 Mar 7;13(3):e0193446. doi: 10.1371/journal.pone.0193446 (PMC5841791; doi:10.1371/journal.pone.0193446)
Supplement: S3 Fig — (PDF) [file pone.0193446.s005.pdf]

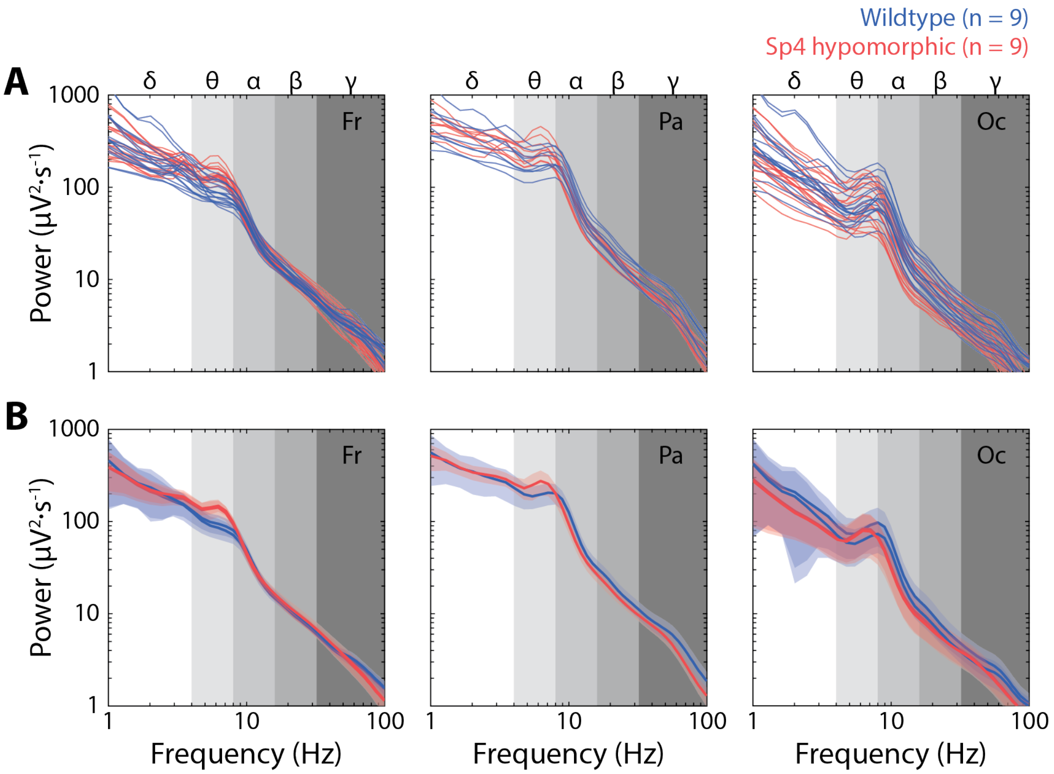

Figure S3. Power spectra of frontal, parietal and occipital recording sites. (A) Each thing line is the power spectra of s single animal, genotype color-coded, both sides pooled together where data available. (B) Population statistics, i.e. mean and standard deviation, of power spectra.
