# Supplementary material for: Ketamine independently modulated power and phase-coupling of theta oscillations in Sp4 hypomorphic mice
Source: PLoS One. 2018 Mar 7;13(3):e0193446. doi: 10.1371/journal.pone.0193446 (PMC5841791; doi:10.1371/journal.pone.0193446)
Supplement: S4 Fig — (PDF) [file pone.0193446.s006.pdf]

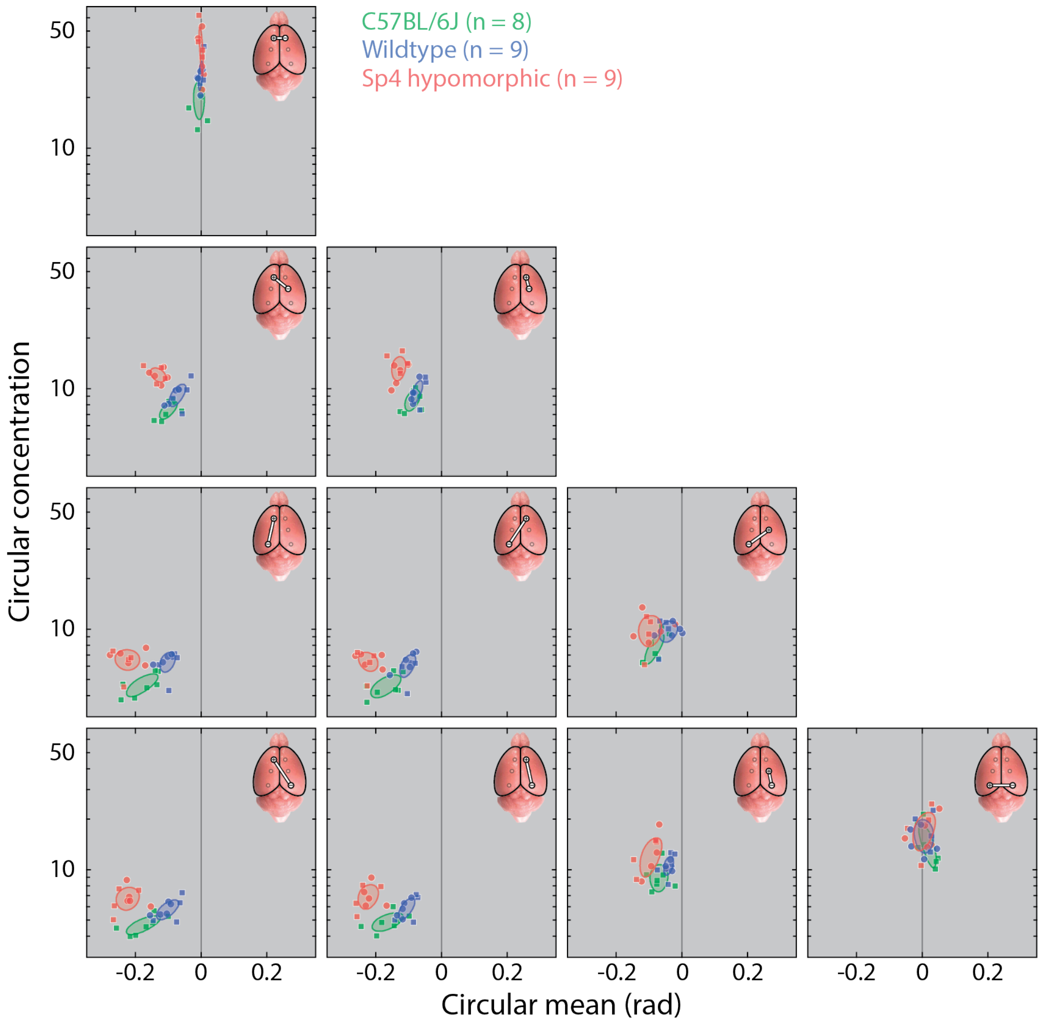

Figure S4. **Magnitude of cortical theta phase progression is dependent on genetic background.** Each panel in this figure is a scatter plot of the first- against the second-order descriptive statistics (i.e. circular mean and concentration) of the theta phase differences between a pair of cortical sites, specified by the inset in the top-right corner. Each symbol represents an animal, square for males and circles for females. Three groups are shown (in different colors): C57BL/6J mice (green), wildtype (blue) and *Sp4* hypomorphic (red) animals. Ovals mark 1-σ contours of covariances.
