# Supplementary material for: Ketamine independently modulated power and phase-coupling of theta oscillations in Sp4 hypomorphic mice
Source: PLoS One. 2018 Mar 7;13(3):e0193446. doi: 10.1371/journal.pone.0193446 (PMC5841791; doi:10.1371/journal.pone.0193446)
Supplement: S5 Fig — (DOCX) [file pone.0193446.s007.docx]

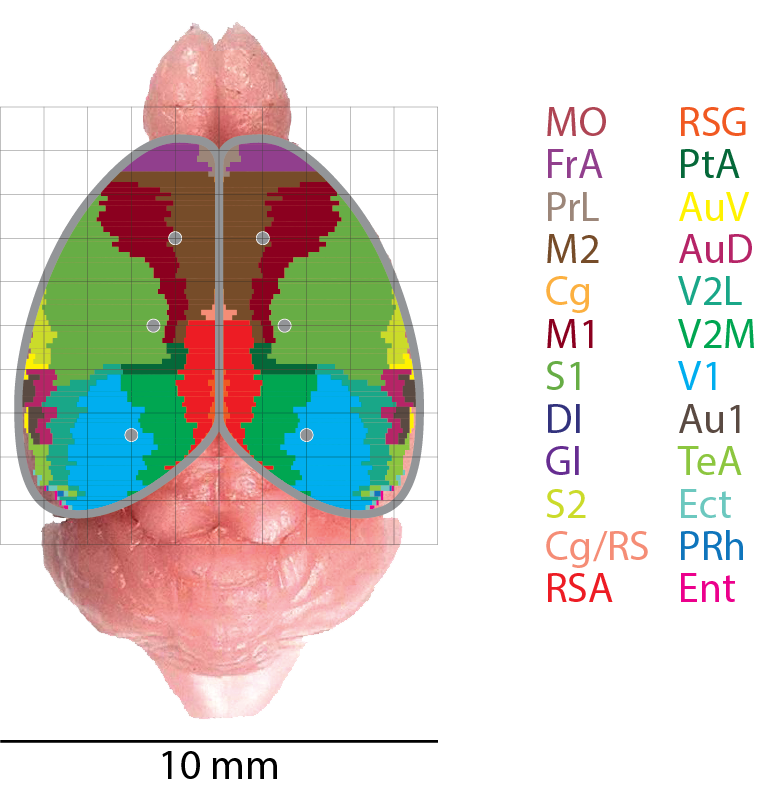


Figure S5. Functional cortical regions corresponding to the epidural recording sites. We reconstructed the functional cortical map (colored regions) on a scale-normalized mouse brain, based on the Paxinos-Franklin mouse brain atlas [1]. The epidural recording sites chosen in the present study are marked on top of the map as gray dots. Legend specifies cortical regions, abbreviations consistent with the Paxinos-Franklin atlas. The Oc sites are within V1, the Pa sites within S1 and the Fr sites on the border between M1 and M2.
